# Dynamic expression analysis of peripheral blood derived small extracellular vesicle miRNAs in sepsis progression

**DOI:** 10.1111/jcmm.18053

**Published:** 2023-11-28

**Authors:** Xinyan Liu, Dapeng Yu, Tiantian Li, Kehan Zhu, Yang Bi, Chaofan Wang, Chunting Wang, Xuan Song

**Affiliations:** ^1^ ICU, Dong E Hospital Liaocheng China; ^2^ Cardiac Surgery Department Dong E Hospital Liaocheng China; ^3^ High Dependency Unit Shandong Public Health Clinical Center Jinan China; ^4^ Shandong First Medical University Jinan China; ^5^ ICU, Shandong Provincial Hospital Affiliated to Shandong First Medical University Jinan China; ^6^ Endocrine and Metabolic Diseases Hospital of Shandong First Medical University Jinan China

**Keywords:** biomarker, immune disorders, miRNA, sepsis, small extracellular vesicle

## Abstract

Immune disorders caused by sepsis have recently drawn much attention. We sought to dynamically monitor the expression of small extracellular vesicle (sEV) miRNAs in peripheral blood during sepsis to explore these miRNAs as potential biomarkers for monitoring immune function in sepsis patients. This study included patients with sepsis. Blood samples were obtained from 10 patients on the first through 10th days, the 12th day and the 14th day since sepsis onset, resulting in 120 collected samples. Serum sEVs were extracted from peripheral venous blood, and levels of MIR497HG, miR‐195, miR‐497, and PD‐L1 in serum sEVs were detected by qPCR, and clinical information was recorded. Our study revealed that the levels of MIR497HG, miR‐195, miR‐497 and PD‐L1 in serum sEVs showed periodic changes; the time from peak to trough was approximately 4–5 days. The levels of sEV MIR497HG and miR‐195 had a positive linear relationship with SOFA score (*r* values were −0.181 and −0.189; *p* values were 0.048 and 0.039, respectively). The recorded quantities of sEV MIR497HG, miR‐195 and PD‐L1 showed a substantial correlation with ARDS. ROC curve analysis revealed that sEV MIR497HG, miR‐195 and miR‐497 could predict the 28‐day mortality of sepsis patients with an AUC of 0.66, 0.68 and 0.72, respectively. Levels of sEVs MIR497HG, miR‐195, miR‐497 and PD‐L1 showed periodic changes with the immune status of sepsis, which provides a new exploration direction for immune function biomarkers and immunotherapy timing in sepsis patients.

## 1 INTRODUCTION

The prototypical presentation of Sepsis is critical organ dysfunction. This dysfunction results from a poorly‐regulated host immune response to infection: a major cause of death in critical patients worldwide.^1^ There are an estimated 48.9 million cases of sepsis and 11.0 million sepsis‐related deaths each year worldwide.^2^ Sepsis is a syndrome often characterized by high heterogeneity—a result of its complex pathophysiological mechanism. The heterogeneity of sepsis patients includes both the range of infection characteristics and the immune status of each patient at any point in time.^3^ Therefore, clinicians face marked challenges in identification, immune monitoring and delivery of treatment in regard to patients with sepsis. The previously stated challenges demonstrate an urgent need for the discovery of a biomarker to monitor immune status in septic patients.

One extensively investigated checkpoint regulator in the domain of sepsis is programmed cell death‐1 (PD‐1).^4^ The regulation of PD‐1 expression involves various upstream markers that contribute to its modulation in different contexts. PD‐1 has been identified as a marker for T‐cell exhaustion during viral infections, suggesting that some exhausted CD4^+^ PD‐1^+^ T cells may convert into induced regulatory T cells (iTregs) upon stimulation through the PD‐L1: PD‐1 pathway.^5^ The stimulation of PD‐1 in T cells is shown to provoke the release of immunosuppressive molecules, possibly culminating in apoptosis. Enhanced T cell expression of PD‐1 in peripheral blood was associated with reduced T cell proliferative capacity, increased nosocomial infection incidence and increased sepsis patient mortality.^6^ Of note, the modification of PD‐1 expression may predict a phenomenon of ‘immunoparalysis’ capable of affecting the prognosis of patients. miRNA is a category of non‐protein‐coding RNA periodically involved in cell proliferation, apoptosis, differentiation, senescence and other cellular processes. In certain cancers, such as pancreatic cancer and lymphoma, PD‐L1 may be targeted and regulated by miR‐195‐5p.^7^, ^8^, ^9^ miR‐195 is encoded by the long non‐coding RNA MIR497HG of its precursor host gene. According to the gene element structure shown by NCBI, both the expression of miR‐195 and that of miR‐497 may be coupled from MIR497HG. It is clear that miR‐497 and miR‐195 may simultaneously regulate PD‐L1 and inhibit its expression. However, the complex mechanism of these miRNAs in sepsis remains unclear.

Small extracellular vesicles (sEV) play a critical role in the body's inflammatory response,^10^ coagulation processes^11^ and cardiac dysfunction.^12^ These factors contribute vitally to the various pathophysiologies of organ dysfunction in sepsis. Studies have shown that secreting tumour growth factor β1 through sEVs may regulate anti‐inflammatory effects.^13^ Additionally, sEVs contain various proteins, including chemokines and inflammatory cytokines—tumour necrosis factor (TNF) ‐α, IL‐1β, CXCL2 and CXCL8, for example—which perform important immunomodulatory functions. Park et al. reported high expression of PD‐L1 and PD‐L2 in circulating sEVs detected in plasma taken from sepsis patients, indicating that plasma‐derived sEVs may also lead to immunosuppression.^14^

In this study, the levels of serum sEVs MIR497HG, miR‐195, miR‐497 and PD‐L1 in sepsis patients were sought in order to identify any connection between these sEVs and clinical disease evolution as measured by SOFA scores. Furthermore, detection of MIR497HG, miR‐195, miR‐497 and PD‐L1 permitted the exploration of novel biomarkers of immune function in sepsis, providing new ideas for precise immunotherapy.

## 2 MATERIALS AND METHODS

We diagnosed sepsis using The Third International Consensus Definitions for Sepsis and Septic Shock (Sepsis‐3).^15^ Sepsis patients admitted to the ICU of Dong E Hospital from January 2022 to December 2022 were included. Other inclusion specifications included age ≥18 years and informed consent. We excluded patients taking immunosuppressive drugs as well as immunocompromised patients with a history of stem cell transplantation. Additionally, patients with neutropenia, patients with current hematologic malignancies, patients with an unknown time of sepsis onset and patients who had spent more than 24 h in the ICU at the time of the required data collection point were also excluded. All aspects of this study were approved by the Review committee of Dong E Hospital.

### 2.1 Sample collection

Blood samples were acquired from sepsis patients within 1 day (24 h) of admission to the ICU. These samples were further grouped according to the time of sepsis onset: the time from the onset of symptoms to the time each blood sample was taken. The time of symptom onset was self‐reported by patients. Blood samples were secured from 10 patients on the first through 10th days, the 12th day and the 14th day. This resulted in a total of 120 samples.

Blood samples were collected by venipuncture or pre‐existing intravascular catheter. Samples were centrifuged at 3000 × g for 15 min, and the serum supernatant was cumulated, divided into equal portions and stored at −80°C. Demographic and clinical information—such as source and type of infection—was attained from electronic medical records of included patients. Other information extracted included Acute Physiology and Chronic Health Evaluation Score (APACHE II) and Sequential Organ Failure Assessment Score (SOFA). Outcome data included ICU length of stay and 28‐day mortality status.

### 2.2 Extraction and identification of serum sEVs

SEVs were separated from 2 mL of serum according to the instructions for sEV separation reagent (Thermo Scientific, Waltham, MA). The extracted sEV suspension of 10 μL was adsorbed to 200 mesh carbon membrane carrier net and negatively stained with uranium dioxyacetate. The morphology of extracted sEVs was identified by transmission electron microscopy, western blot and nanoparticle particle size detection (Appendix S1).

### 2.3 Extracting exosomal RNA and reverse transcription cDNA synthesis

TRIzol reagent was used to withdraw total RNA from serum sEVs. An RNA concentration detector was used to measure the RNA concentration. To synthesize cDNA, we first mixed the RNA template, Primer Mix, dNT PMix, DTT, RT Buffer, HiFiScript and RNase‐Free Water. This mixture was placed on ice for later use. The volumes required for this reaction are included in Table 1. The total reaction volume was 20 μL. Any solution on the wall of the tube after mixing was collected to the bottom of the tube by vortex mixing and temporary centrifugation. At the end of the reaction, the product was centrifuged briefly and placed on ice to cool. The cDNA products were used for qPCR.

**TABLE 1:** qPCR reagent volumes.

Reagent	Quantity added per tube
dNTP mix	4 μL
Primer mix	2 μL
RNA template	2 μL
5 × RT Buffer	4 μL
DTT, 0.1 M	2 μL
HiFiScript, 200 U/μL	1 μL
RNase‐free water	Up to 20 μL

### 2.4 qPCR detection

Quantitative fluorescence PCR was used to quantify the expression inventories of MIR497HG, miR‐195, miR‐497 and PD‐L1 using the standard curve method. Results were calculated and recorded using the 2−∆∆Ct method.

### 2.5 Statistical analysis

SPSS 22.0 statistical software was utilized to conduct all statistical analyses. For baseline characteristics of sepsis patients, continuous variables conforming to a normal distribution were conveyed as mean ± standard deviation, and categorical variables were indicated as percentages. The levels of miRNAs were compared between time since onset groups using the Wilcoxon rank sum test. Scatter plots were used to draw fitting curves to analyse the dynamic changes of sEVs MIR497HG, miR‐195, miR‐497 and PD‐L1 at different onset times. Associations between sEVs—MIR497HG, miR‐195, miR‐497 and PD‐L1—and acuteness of organ failure, as determined by SOFA score, were investigated using the linear regression. Spearman's correlation analysis was employed to evaluate the relationship between levels of sEVs MIR497HG, miR‐195, miR‐497 and PD‐L1, with ARDS and AKI. We performed a receiver operating characteristic (ROC) analysis to evaluate the predictive value of sEV level as prognostic predictors. *p* < 0.05 was considered statistically significant.

## 3 RESULTS

One hundred twenty sepsis samples were included according to the time of sepsis onset. These samples were collected from 10 patients on each day from the first through 10th, 12th and 14th day since sepsis onset. Among these cases, 76 (63.3%) were male. Common complications included cardiovascular disease, chronic lung disease, diabetes and cerebrovascular disease. 34 patients (28.3%) developed septic shock, and the infection sites observed were primarily lung and abdominal. 39 patients (31.7%) died within 28 days. Patient characteristics are displayed in Table 2.

**TABLE 2:** Patient characteristics.

	All patients (*n* = 120)	Survival group (*n* = 81)	Death group (*n* = 39)	*t*/χ^2^	*p*‐value
Male sex, *n* (%)	76 (63.3%)	48 (59.3%)	28 (71.8%)	1.781	0.182
Age, years	67.53 ± 14.78	66.31 ± 15.70	70.05 ± 12.48	1.697	0.195
Body mass index, kg/m^2^	23.62 ± 3.27	23.46 ± 3.53	23.961 ± 2.66	0.612	0.436
Comorbidity, *n* (%)					
Cardiovascular disease	38 (31.7%)	22 (27.2%)	16 (41.0%)	2.339	0.126
Chronic Pulmonary Disease	13 (10.8%)	7 (8.6%)	6 (15.4%)	1.239	0.266
Diabetes mellitus	30 (25.0%)	20 (24.7%)	10 (25.6%)	0.013	0910
Cerebrovascular disease	35 (29.2%)	18 (22.2%)	17 (43.6%)	5.818	0.016
Immune system disease	3 (2.5%)	0 (0)	3 (7.7%)	6.391	0.011
Chronic liver disease	2 (1.7%)	2 (2.5%)	0 (0)	0.979	0.322
Chronic kidney disease	2 (1.7%)	1 (1.2%)	1 (2.6%)	0.284	0.594
Tumour	8 (6.7%)	8 (9.9%)	0 (0)	4.127	0.042
APACHEII^a^, median (IQR)	17 (12, 21.75)	16 (11, 20.5)	20 (14,25)	2.654	0.008
SOFA^b^, median (IQR)	5 (4, 7)	5 (3,7)	6 (4,9)	2.298	0.022
Septic shock, *n* (%)	34 (28.3%)	20 (26.0%)	14 (35.9%)	1.230	0.267
Source of infection, *n* (%)				5.880	0.118
Pneumonia	68 (56.7%)	40 (49.5%)	28 (71.8%)		
Abdomen	37 (30.8%)	30 (37.0%)	7 (17.9%)		
Urinary tract	9 (7.5%)	7 (8.6%)	2 (5.1%)		
Other	6 (5.0%)	4 (4.9%)	2 (5.1%)		
Blood tests at ICU^c^ admission					
White blood cell count (×10^9^/L)	12.02 ± 7.11	10.94 ± 6.24	14.27 ± 8.29	6.025	0.016
Neutrophil count (×10^9^/L)	10.63 ± 6.59	9.62 ± 5.81	12.73 ± 7.62	6.123	0.015
Granulocyte (%)	86.63 ± 8.26	85.63 ± 8.74	88.69 ± 6.81	3.704	0.057
Lymphocyte count (×10^9^/L)	0.73 ± 0.57	0.70 ± 0.53	0.81 ± 0.64	0.913	0.341
Lymphocyte (%)	7.52 ± 5.90	7.97 ± 6.20	6.58 ± 5.19	1.449	0.231
Requirement for vasopressor	50 (41.7%)	36 (44.4%)	14 (35.9%)	0.791	0.374
Requirement for mechanical ventilation	63 (52.5%)	46 (56.8%)	17 (43.6%)	1.839	0.175
Requirement for CRRT^d^	31 (26.1%)	19 (23.8%)	12 (30.8%)	0.671	0.413
ICU length of stay, days	14.75 ± 20.09	16.07 ± 21.89	18.54 ± 12.06	1.084	0.300
Hospital length of stay, days	17.42 ± 12.74	16.88 ± 13.10	18.54 ± 12.06	0.446	0.506

^a^ Acute Physiology and Chronic Health Evaluation Score.

^b^ Sequential Organ Failure Assessment Score.

^c^ Intensive care unit.

^d^ Continuous renal replacement therapy.

**FIGURE 1:**
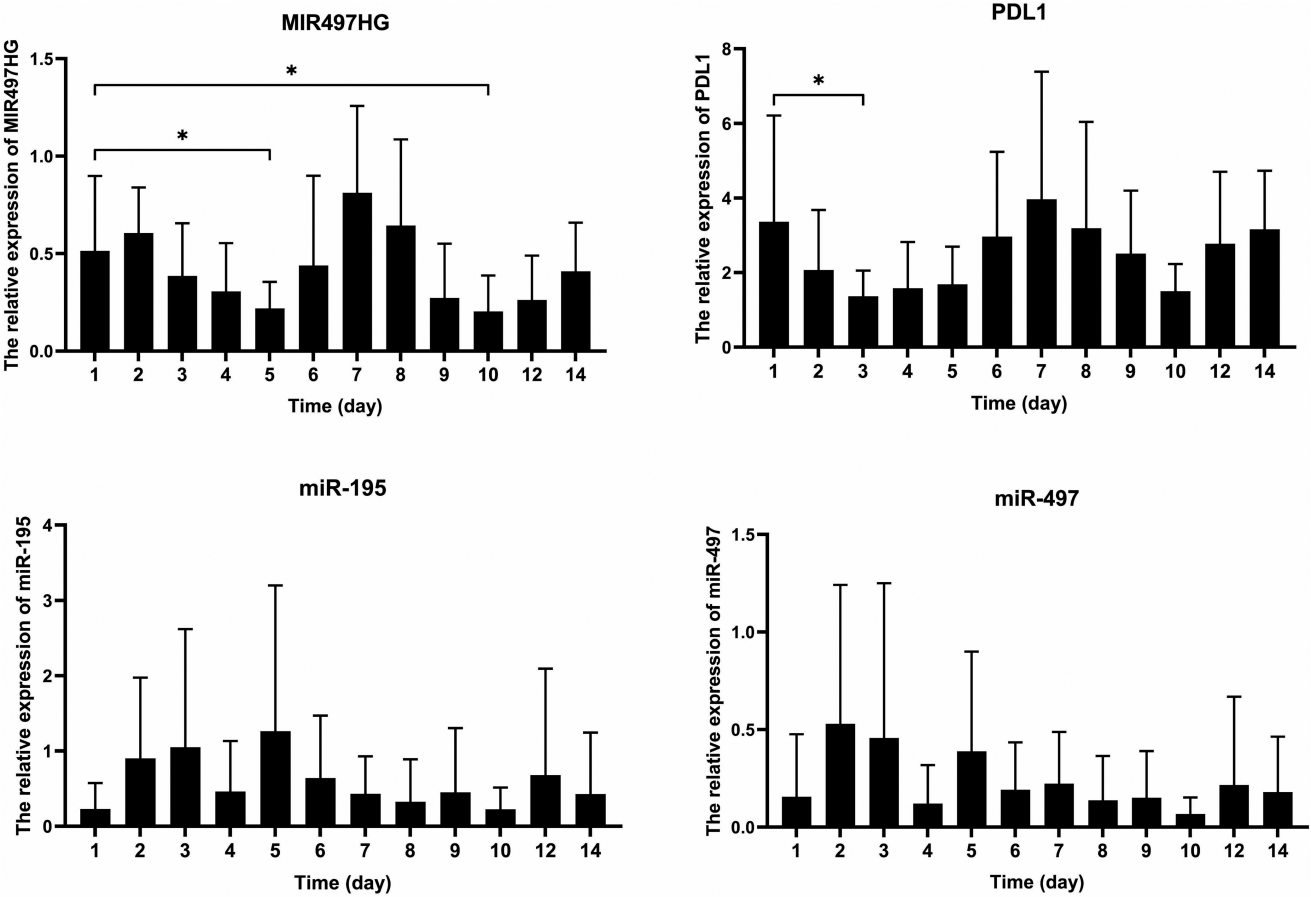
Expression levels of MIR497HG, miR‐195, miR‐497 and PD‐L1 in serum sEVs were quantitatively determined by qPCR. *p < 0.05.

**FIGURE 2:**
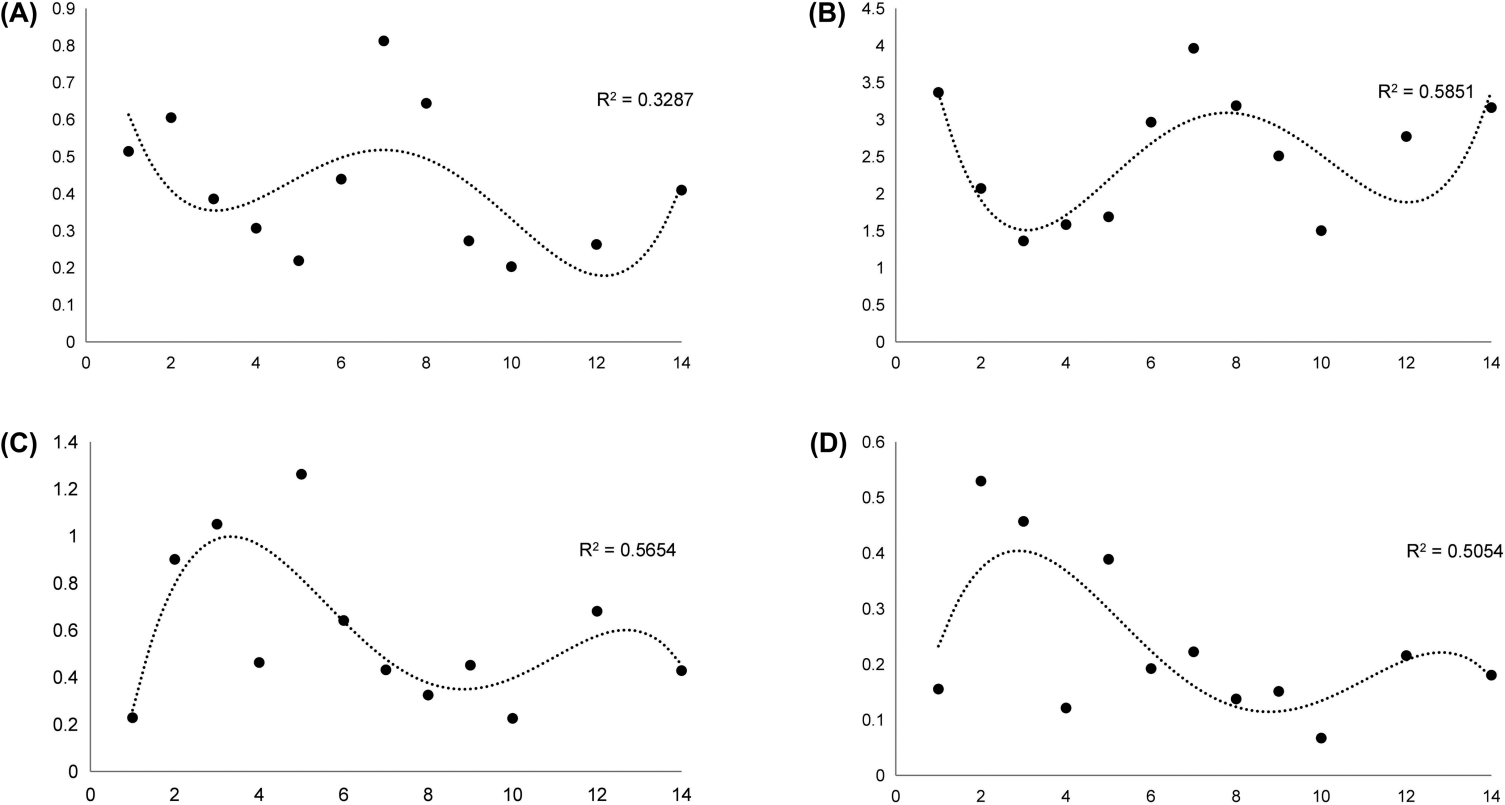
Expression levels by qPCR of exosomal MIR497HG, miR‐195, miR‐497 and PD‐L1 from blood of sepsis patients. (A) Expression levels by qPCR of exosomal MIR497HG; (B) Expression levels by qPCR of exosomal PD‐L1; (C) Expression levels by qPCR of exosomal miR‐195; (D) Expression levels by qPCR of exosomal miR‐497.

Levels of exosomal MIR497HG, miR‐195, miR‐497 and PD‐L1 were measured by qPCR. Results showed the level of sEV miR497HG to be significantly different on Day 1 from Day 5 and Day 10 (*p* < 0.05). The level of sEV PD‐L1 varied significantly between Day 1 and 3 (*p* < 0.05), but had no significant difference with other days (*p* > 0.05). There were no significant differences between the levels of sEVs miR‐195 and miR‐497 on Day 1 and other days (*p* > 0.05) (Figure 1). Further curve fitting analysis showed that the numbers of sEVs MIR497HG, miR‐195, miR‐497 and PD‐L1 displayed periodic changes with different stages of immunoactivation or immunosuppression. The time from peak to trough was approximately 4–5 days (Figure 2).

**FIGURE 3:**
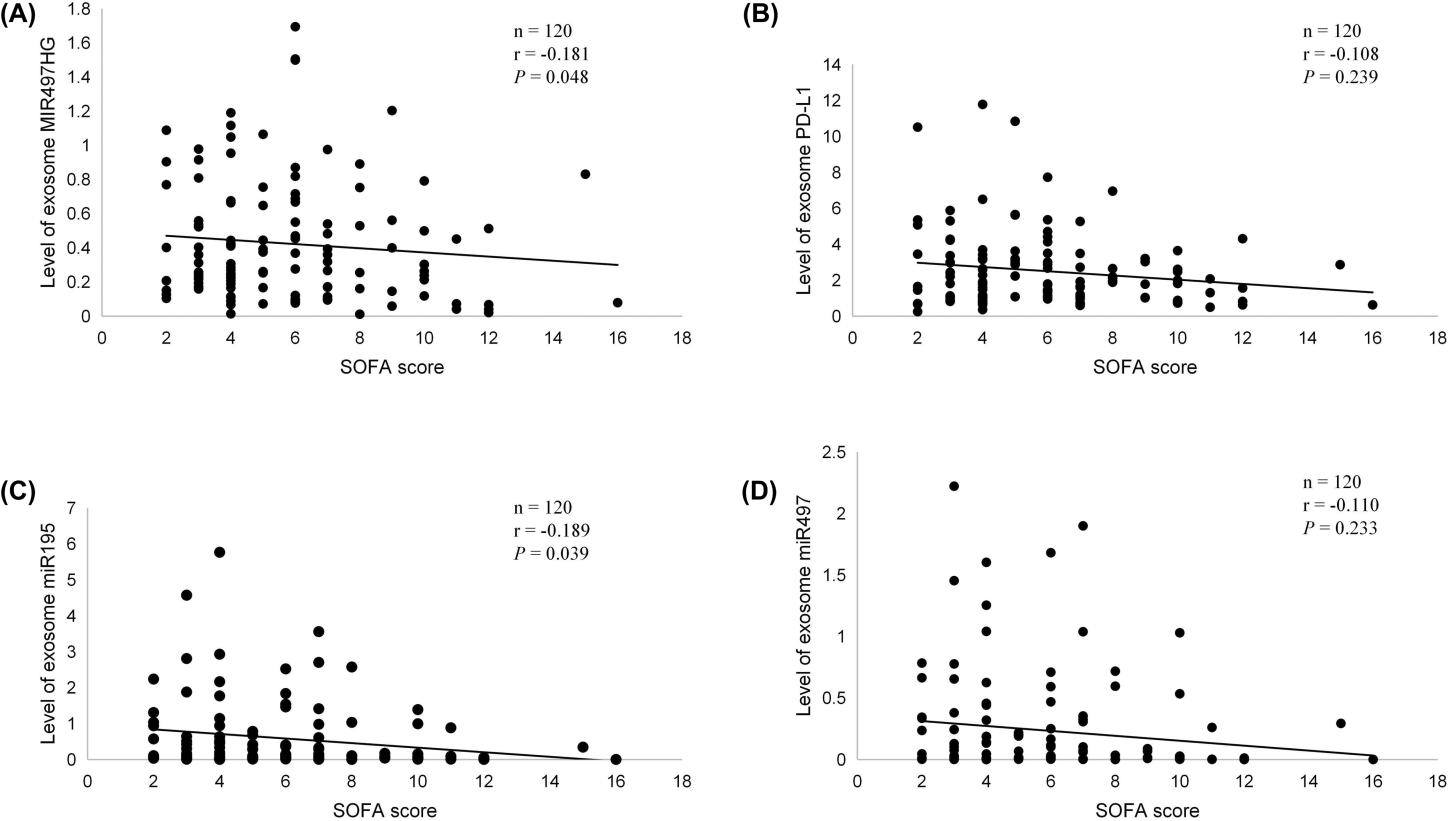
Correlation between exosomal MIR497HG, miR‐195, miR‐497 and PD‐L1 levels and Sequential Organ Failure Assessment (SOFA) score in patients with sepsis.

Associations between sEVs MIR497HG, miR‐195, miR‐497 and PD‐L1 and severity of organ failure were evaluated using the linear regression. In the study cohort, the levels of sEV MIR497HG and those of miR‐195 had a positive linear relationship with SOFA score (*r* values were −0.181 and −0.189; *p* values were 0.048 and 0.039, respectively). The levels of sEVs miR‐497 and PD‐L1 did not have a positive linear relationship with SOFA scores (*r* values were −0.110 and −0.108; *p* values were 0.233 and 0.239, respectively) (Figure 3).

Spearman's correlation analysis was used to evaluate the correlation between the levels of sEVs MIR497HG, miR‐195, miR‐497 and PD‐L1 with ARDS and AKI. Results showed that the levels of sEV MIR497HG, miR‐195 and PD‐L1 were significantly correlated with ARDS, whereas the level of miR‐497 was not correlated with ARDS. The levels of MIR497HG, miR‐195, miR‐497 and PD‐L1 were not correlated with AKI (Table 3).

**TABLE 3:** Correlation between the levels of sEV MIR497HG, miR‐195, miR‐497, PD‐L1 and ARDS, AKI.

	MIR497HG	miR‐195	miR‐497	PD‐L1
ARDS	−0.275*	−0.331*	−0.146	0.184*
AKI	−0.067	−0.076	0.010	−0.115

* *p* < 0.05.

**FIGURE 4:**
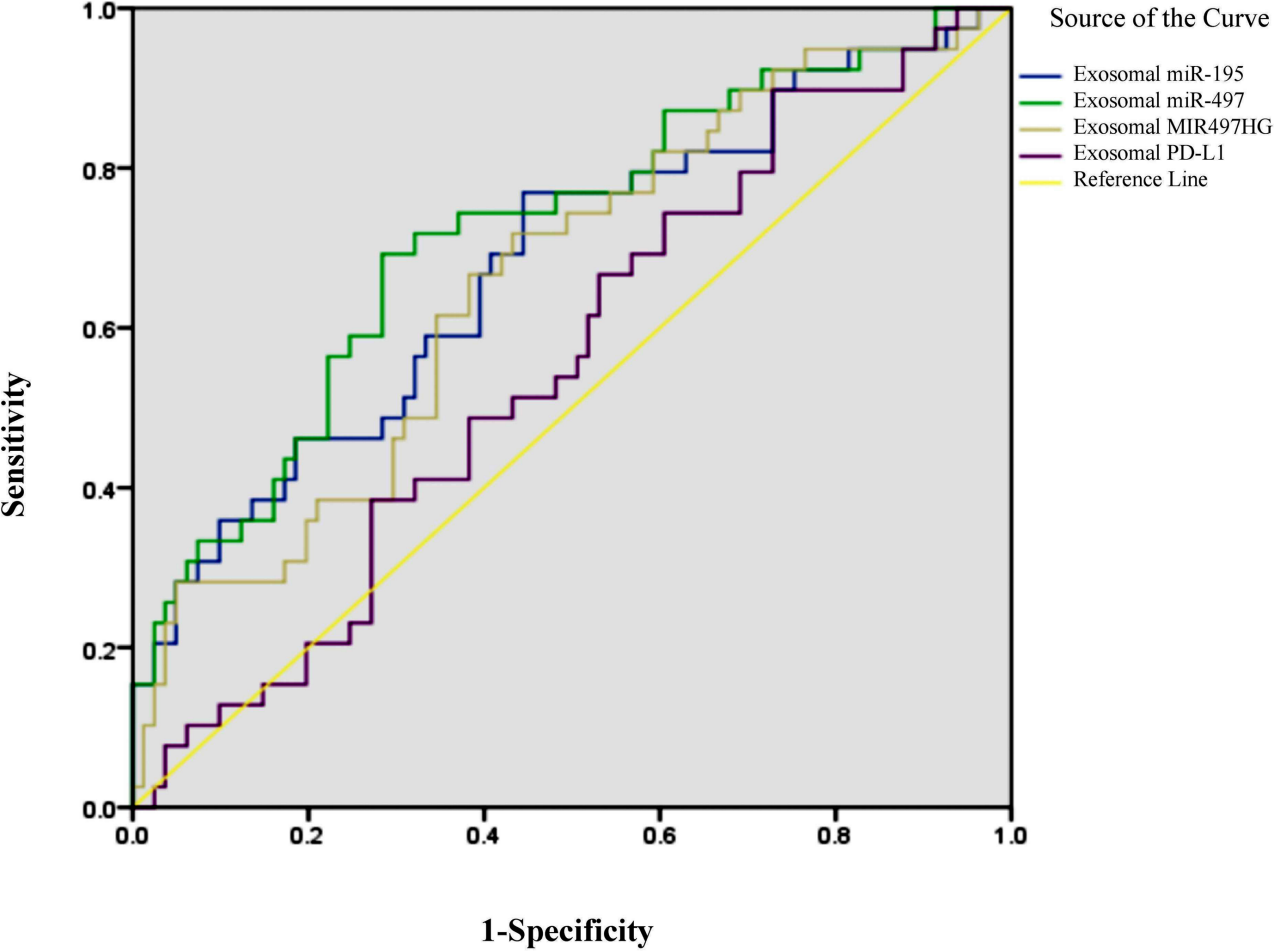
ROC curve for predicting 28‐day mortality of sepsis patients with exosomal MIR497HG, miR‐195, miR‐497 and PD‐L1 levels.

The receiver operating characteristic curve (ROC) was applied to determine the predictive value of sEV levels as prognostic predictors (Figure 4). Results showed that the levels of sEV MIR497HG, miR‐195 and miR‐497 could predict the 28‐day mortality of sepsis patients with an AUC of 0.66 [95% confidence interval (CI) 0.56–0.76], 0.68 [95% CI 0.58–0.79], 0.72 [95% CI 0.62–0.92], respectively. All *p* values were less than 0.05.

## 4 DISCUSSION

In this study, we assessed the dynamic changes of sEV miRNAs that occur during sepsis progression. Concurrently, we investigated the relationship between sEV miRNAs and the corresponding graveness of organ failure and mortality. Results from this study indicated that the levels of sEVs MIR497HG, miR‐195, miR‐497 and PD‐L1 in sepsis patients changed periodically with the progression of sepsis; the time from peak to trough was approximately 4–5 days. We also found that the levels of sEV MIR497HG and miR‐195 were associated with the acuteness of organ failure, and the levels of sEV MIR497HG, miR‐195 and miR‐497 were correlated with a 28‐day mortality rate in patients with sepsis.

Sepsis coincides with a high inflammatory response and immunosuppression, but the specific changes in immune status are not well understood.^16^ Recent studies in knockout (KO) mice clearly demonstrate the mechanistic role that miR‐146a and the single‐stranded RNA sensor TLR7 play in sepsis‐induced inflammation and organ injury.^17^, ^18^ As evidenced by preceding studies, exosomal contents, such as miRNA or proteins from diverse origins, can arouse or inhibit myocardial dysfunction, acute lung injury and acute kidney injury.^19^, ^20^, ^21^ A mouse model displayed an association between exosomal microRNA (miR‐126) derived from endothelial progenitor cells, and microvascular dysfunction.^20^ Furthermore, Gurien et al. found that ex‐miR‐130b‐3p specifically binds to the extracellular cold‐inducible RNA binding protein, CIRP, a known proinflammatory DAMP in sepsis. Ex‐miR‐130b‐3p decreases its binding affinity to TLR4/MD2 and attenuates CIRP‐induced inflammation.^22^ Together, these investigations demonstrate the importance of sEV miRNAs in innate immunity regulation while simultaneously highlighting the complexity of sEV function.

The levels of circulating miR‐15 are significantly increased in septic patients and may be linked to septic death. Zheng et al. found that the pronouncement of miRNA‐195 in lung and liver tissues of sepsis model mice was significantly increased.^23^ Furthermore, silencing miRNA‐195 by gene KO can increase the expression of certain proteins—BCL‐2, Sirt1 and Pim‐1—and significantly reduce the potential trans‐organ damage associated with sepsis. This silencing can advance the overall survival rate of sepsis model mice. Yuan et al. conducted a similar experiment, performing a microarray analysis of cecal‐ligation‐induced sepsis animal models. This experiment revealed the upregulation of miR‐195 in sepsis cell models as well as the key role of miR‐195 in endorsing apoptosis of intestinal epithelial cells. The upregulation of miR‐195 leads to the exacerbation of sepsis by its own targeting of SIRT1, thus promoting apoptosis of intestinal epithelial cells.^24^ The study showed that miR‐195 was encoded by the long non‐coding RNA—MIR497HG—of its precursor host gene. According to the NCBI gene element structure, the pronouncement of miR‐195 and miR‐497 alike may both be coupled from MIR497HG.^25^ Furthermore, miR‐497 and miR‐195 can simultaneously target PD‐L1 and inhibit its expression. PD‐L1, as a co‐stimulant and co‐inhibitor, also performs regulatory functions in regard to adaptive and innate immune responses.^26^ This study showed that the levels of sEVs MIR497HG, miR‐195, miR‐497 and PD‐L1 in sepsis patients exhibited periodic changes with different stages of immunoactivation and immunosuppression; the time from peak to trough was approximately 4–5 days. These data suggest that MIR497HG may affect the transport pattern of PD‐L1. MIR497HG is expected to be a biomarker for monitoring immune function during sepsis, thus aiding in the application of precise immunotherapy.

Exosomal miRNAs can also be used as negative regulators of inflammatory cells.^27^, ^28^, ^29^ Kyeongman Jeon et al. sought to determine if sEV levels were associated with organ failure and mortality through assessing the overall levels of plasma sEVs in sepsis patients.^30^ They discovered a trend of increased sEV levels in control, sepsis patient and septic shock patient groups (204 μg/mL vs. 525 μg/mL vs. 802 μg/mL, *p* < 0.001). Additionally, a positive linear relationship was noted between overall sEV levels and SOFA score in the study cohorts (*r* value = 0.47). Patients were divided into two groups—according to best cut off level—and a statistical difference in 28 and 90‐day mortality between patients with high and low plasma sEVs was detected. This study showed that elevated sEV levels were associated with an increased severity of organ failure and were predictive of mortality in critically ill sepsis patients. In this study, overall plasma sEV levels in sepsis patients were rated without specific miRNAs analysis. We evaluated the correlation between the levels of sEVs MIR497HG, miR‐195, miR‐497 and PD‐L1 and severity of organ failure and mortality. We found that the levels of sEVs MIR497HG and miR‐195 were correlated with the severity of organ failure. The levels of sEV MIR497HG, miR‐195 and miR‐497 were associated with 28‐day mortality in sepsis patients. However, no relationship was observed between PD‐L1 and ARDS, or AKI and 28‐day mortality. Therefore, the role of sEV miRNAs in immunosuppression and organ damage induced by sepsis warrants further study.

In the present investigation, we comprehensively assessed the dynamics of sEV miRNAs in different stages of sepsis progression. We did this while evaluating the relationship between sEV miRNAs and magnitude of organ failure and mortality in a large sepsis cohort. However, our study had several limitations. First, the research was conducted at a single referral center, possibly limiting the generalizability of the data. Second, patients were grouped according to self‐reported sepsis onset time, which may have led to grouping inaccuracies. Third, due to the retrospective nature of this study, we were unable to include relevant clinical immune indicators for statistical analysis.

## 5 CONCLUSIONS

The levels of sEVs MIR497HG, miR‐195, miR‐497 and PD‐L1 showed periodic changes in immune status in patients suffering from sepsis. The levels of sEVs MIR497HG and miR‐195 correlated with the severity of organ failure. The levels of sEVs MIR497HG, miR‐195 and miR‐497 were associated with 28‐day mortality in sepsis patients. These data are expected to yield new biomarkers for monitoring immune function during sepsis, in addition to the opportunity to explore new directions in the study of the sepsis immunotherapy.

## AUTHOR CONTRIBUTIONS

**Xinyan Liu:** Data curation (equal); writing – original draft (equal). **Dapeng Yu:** Data curation (equal); writing – original draft (equal). **Tiantian Li:** Data curation (equal); writing – original draft (equal). **Kehan Zhu:** Formal analysis (equal); methodology (equal). **Yang Bi:** Formal analysis (equal); methodology (equal). **Chaofan Wang:** Formal analysis (equal); methodology (equal); software (equal). **Chunting Wang:** Writing – review and editing (equal). **Xuan Song:** Conceptualization (equal); funding acquisition (equal); project administration (equal); supervision (equal); writing – review and editing (equal).

## FUNDING INFORMATION

This work was supported by National Natural Science Foundation of China (Grant 82102274) and Natural Science Foundation of Shandong Province, China (Grant ZR202102210272), Taishan Scholars Program of Shandong Province (Grant tsqn202211347), Shandong Provincial Postdoctoral Science Foundation (Grant SDCX‐ZG‐202202025) and Sepsis Translational Medicine Laboratory of Liaocheng City (Grant N/A). The funders did not and will not have a role in study design, data collection and analysis, decision to publish, or preparation of the manuscript.

## CONFLICT OF INTEREST STATEMENT

The authors declared no conflicts of interest concerning the research, authorship, and/or publication of this article.

## Supporting information


Appendix S1.

## Data Availability

The data supporting the findings of this study are available upon reasonable request from the corresponding author.
