# Supplementary material for: Dynamic expression analysis of peripheral blood derived small extracellular vesicle miRNAs in sepsis progression
Source: J Cell Mol Med. 2023 Nov 28;28(2):e18053. doi: 10.1111/jcmm.18053 (PMC10826429; doi:10.1111/jcmm.18053)
Supplement: Supplementary file 1 — Appendix S1. [file JCMM-28-e18053-s001.docx]

**Supplemental 1**. **Extraction and identification of serum exosomes**


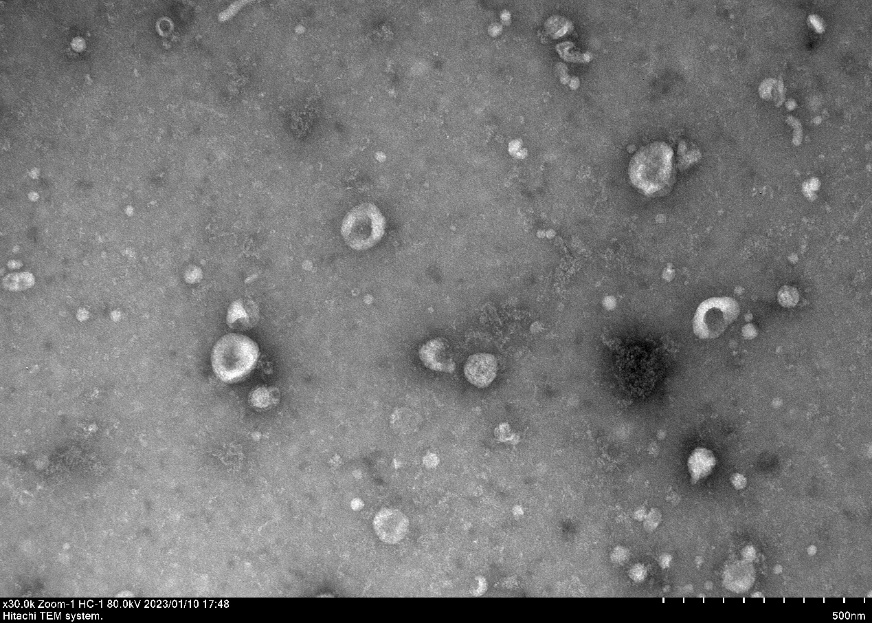


**Figure 1.** **Electron microscopy detection results of exosomes.**


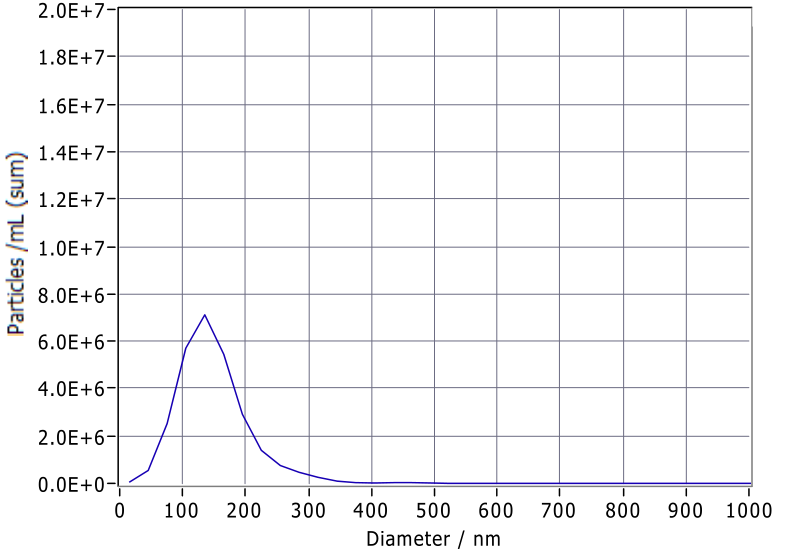


**Figure 2. Exosome particle size results.**

**
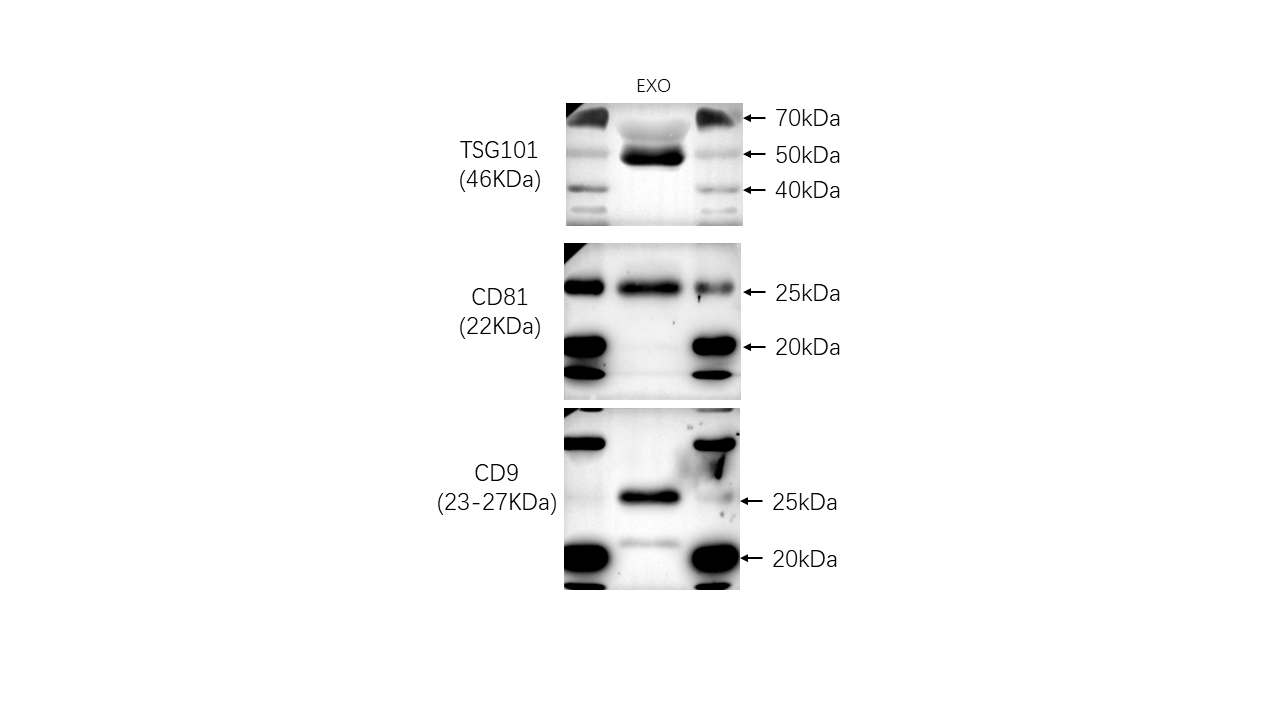
**

**Figure 3. Western Blot to detect TSG101, CD81 and CD9 tag proteins in exosomes.**
